# Factors Associated with Antiretroviral Therapy Re-Engagement Among Men Who Have Sex with Men in South Africa: A Multi-District Analysis

**DOI:** 10.3390/v18050539

**Published:** 2026-05-07

**Authors:** Betty Sebati, Anthony Brown

**Affiliations:** School of Interdisciplinary Research and Graduate Studies, College of Graduate Studies, University of South Africa, Pretoria 0002, South Africa; browna@unisa.ac.za

**Keywords:** men who have sex with men, antiretroviral therapy, re-engagement, South Africa

## Abstract

Men who have sex with men (MSM) are among of the key population groups that have been disproportionately affected by the HIV epidemic globally. Hence, MSM living with HIV may experience unique challenges leading to their disengagement from and re-engagement with care. This study aimed to identify factors associated with ART re-engagement among MSM in selected districts of South African provinces. A retrospective observational study design was followed, utilising MSM routine programme data from 1 January 2018 to 31 December 2022. The programme enrolled 3337 MSM aged 16 years or older who resided in the selected provinces/districts. Descriptive statistics characterised participants’ baseline profiles. Binary logistic regression identified factors associated with re-engagement with ART. Data analysis was done using SPSS version 31.0.1.0; *p* < 0.05 was considered statistically significant. The district was the only statistically significant predictor of re-engagement among MSM, wherein eThekwini district had lower odds of re-engagement (aOR = 0.248, 95% CI: 0.144–0.428, *p* < 0.001). This represented a 75% reduction in the likelihood of re-engagement compared to the City of Tshwane. There was no association between being re-initiated on ART and HIV testing modality (aOR = 7.299, 95% CI: 0.567–94.037, *p* = 0.127). Future studies should longitudinally and qualitatively investigate specific programme and contextual factors driving district-level variation in re-engagement, while incorporating individual-level factors.

## 1. Introduction

Men who have sex with men (MSM), including gay, bisexual, as well as other MSM who engage in same-sex behaviours, are part of the key population groups that have been disproportionately affected by the HIV epidemic globally [1]. This is attributed to discrimination, stigma, and marginalisation worldwide, as well as criminalisation in some countries such as Senegal, Ghana and Mali [2]. Other key population groups include sex workers, people who inject drugs, transgender people, and prisoners with global HIV prevalences of 2.7%, 7.1%, 8.5%, and 1.4%, respectively [2]. Specifically, MSM account for a global median HIV prevalence of 7.6% [2] and have a 26 times higher risk of HIV acquisition than the general population [1]. Moreover, MSM in sub-Saharan Africa had a prevalence of over 15% in approximately 60 countries, a region that accounts for two-thirds of people living with HIV (PLHIV) in the world [3]. MSM in this region have over 4.9 times higher risk of acquiring HIV than their counterparts in the general population [4].

South Africa continues to be the global epicentre of HIV, with around 8 million people living with HIV, equivalent to approximately 12.7% of the population [2,5]. The prevalence of HIV among MSM in South Africa is estimated to range from 13 to 49%, which is almost one to five times greater than that of their counterparts in the general population [6]. This disproportionately high prevalence is partly attributable to the structural barriers MSM face (i.e., stigma, discrimination, criminalisation), which limit their access to HIV prevention and treatment services [7]. This contributes to gaps resulting from the under-reporting and invisibility of MSM and key populations in HIV surveillance systems and national programmes [8,9].

South Africa has the largest ART programme globally [10,11], and as of 2025, it was reported that 96% of PLHIV knew their status, 79% of those who knew their status were on ART, and 94% of those on ART were virally suppressed [10]. This reflects meaningful, but incomplete progress towards the 95-95-95 UNAIDS goals for ending HIV as a public health threat by 2030. The HIV care continuum provides the fundamental framework through which progress can be measured. It encompasses HIV testing, linkage to care, ART initiation, ART adherence, retention in care, and viral suppression [12]. One of the critical challenges in the continuum is the interruption of treatment, wherein an individual discontinues ART for a defined period following initiation (i.e., 30 days, 60 days, 90 days, etc.) [13]. Such interruptions may be due to several factors, including the inaccessibility of health facilities, stigma and discrimination from the public or within health facilities, psychological barriers such as difficulty accepting an HIV diagnosis, or experiencing treatment fatigue at an individual level, among other factors [14,15,16]. This can lead to several consequences, including viral rebound, disease progression, drug resistance, and even increased HIV transmission. Disengagement from care, therefore, not only compromises the health outcomes of the individual but also affects population health as treatment interruption continues [14].

Addressing the population health impact requires timely re-engagement in care, which is vital to restore and maintain a virally suppressed state, while preventing disease progression and reducing the rate of HIV transmission at both the individual and population levels [17]. Although this is a critical aspect, little research has been done on it compared to other aspects of the HIV care continuum [18], particularly among key populations such as MSM. Re-engagement in care can generally be influenced by several factors, including the presence of friendly healthcare workers in a facility, the implementation of outreach programmes to trace disengaged individuals and encourage treatment continuation [15,16]. The way in which an individual enters the HIV care continuum has implications for their individual’s re-engagement with HIV care after having experienced an interruption [19]. The mode of HIV testing through which an individual receives their initial HIV diagnosis serves as a possible determinant of the individual’s ability to re-engage in HIV care due to how well the method of diagnosis has establishes health system support based on individual needs. Community-based testing strategies such as the Social Network Strategy (SNS) provide navigator services that could facilitate a return to HIV care after an interruption. The SNS is an HIV testing strategy that utilises network connections to refer those at high risk to HIV testing services [20]. However, rapid testing in an emergency department setting provides limited follow-up services after diagnosis [21]. Similarly, the initial linkage can exemplify the level of an individual’s engagement in the healthcare system during the time of entry, which could affect their health-seeking behaviour if they experience interruptions in care at a later stage [22]. For these reasons, both HIV testing modality and linkage were included as predictors of re-engagement with care in the current study. Furthermore, social support, the availability of differentiated service delivery models, various options to access ART, the deterioration of health, or improved health education may similarly facilitate re-engagement, among other factors [15].

This study is guided by the HIV care continuum, which is operationalised through the 95-95-95 UNAIDS goals. Re-engagement with ART is a consequential transition within the continuum, as MSM face disproportionate gaps at each stage that contribute to disengagement from care [15]. These gaps are driven by social and structural barriers, including the dual stigma of simultaneously being MSM and living with HIV, as well as the discriminatory attitudes of healthcare providers, and the scarcity of MSM-friendly HIV services, all of which limit the ability of MSM to access and remain engaged in HIV care [23,24,25]. However, there is limited information on specific contextual barriers in different parts of South Africa. A bio-behavioural study conducted in South Africa reported that ART coverage among MSM within South African cities ranged from 65% in Cape Town to 77% in Johannesburg- [26]. Giovenco et al. [26] identified an urgent need to investigate multilevel determinants of the gaps faced by MSM in HIV care, particularly ART adherence. Despite the increasing recognition of contextual and structural barriers to HIV care among MSM, little is known regarding how programme-level and service delivery variables (i.e., HIV testing modality, linkage pattern, viral load test completion, etc.) relate to ART re-engagement in this population. Understanding these programme-level predictors is vital for targeted service improvements within HIV programmes [27]. Therefore, this study aimed to identify factors associated with ART re-engagement among MSM in selected districts of South Africa.

The study focused on MSM across various districts in three provinces, i.e., Gauteng, Mpumalanga, and KwaZulu-Natal. As the most highly populated and economically active province in South Africa, Gauteng has a considerable number of non-governmental organisations (NGO)-based MSM-related HIV services located within the City of Tshwane and Ekurhuleni, which have adequate healthcare resources in their service delivery [28]. KwaZulu-Natal province bears the greatest burden of HIV in South Africa and is the epicentre of the national HIV epidemic; yet the available service capacity for MSM in the eThekwini and UMgungundlovu districts is deficient when compared to the burden of HIV among key populations/MSM in that region [29,30]. The service delivery infrastructure available to MSM in Mpumalanga is insufficient compared to Gauteng and KwaZulu-Natal. However, when one considers the higher prevalence of HIV-related stigma, MSM in Mpumalanga are likely to seek HIV self-diagnosis and delay the diagnosis until after they have engaged in risky behaviour, and thereby only add to the challenges associated with seeking HIV treatment at clinical sites [26,31]. Together, the differences in provincial HIV service capacity, population density and the burden of living with HIV have created the rationale for the multi-district approach taken in the current study.

## 2. Materials and Methods

### 2.1. Study Design

This study followed a retrospective observational cohort study design, utilising MSM routine programme data from MSM enrolled across five districts in three South African provinces between 1 January 2018, and 31 December 2022. The data were extracted from the MSM programme’s routine monitoring system, which captured each participant’s information at a single time point during enrolment. Each participant was grouped into one of the three ART status groups at enrolment. Each participant contributed one record to the dataset, and changes in ART status over the programme period were not longitudinally tracked.

### 2.2. Sample Size

**Figure 1:**
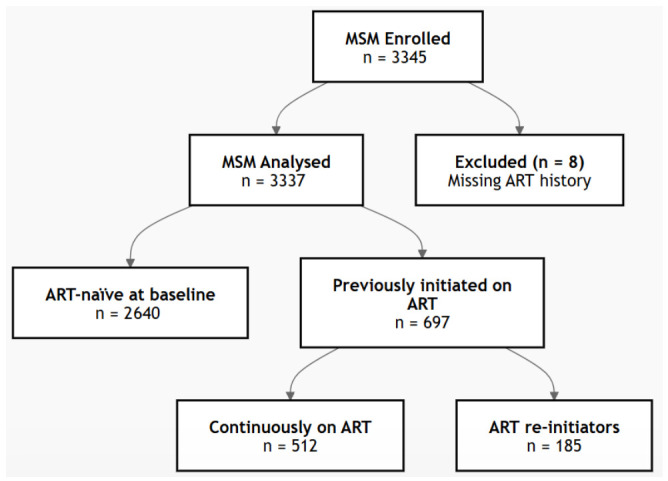
Summary of the sample size.

The programme enrolled 3337 MSM who were 16 years of age and older, who were accessing HIV services from the programme during the study period, and who resided in the selected provinces and districts where the programme was being rolled out. The current study focused on identifying factors associated with ART re-engagement among MSM who re-initiated ART after an ART interruption of ≥60 days. See the summary of the sample size in Figure 1 below:

### 2.3. Study Population and Setting

The MSM programme was implemented in collaboration with the South African Department of Health through its primary healthcare facilities for better reach of vulnerable populations through the provision of HIV, tuberculosis (TB), and sexually transmitted infection (STI) services. The main rationale for the programme and its associated services was the reduction of new infections, as well as contributing towards ending HIV as a pandemic.

The programme operated among MSM, including gay, bisexual, and other MSM who self-reported engaging in same-sex behaviour at programme enrolment; they were aged 16 years or older, and resided within one of the selected districts/provinces of South Africa. The five districts included: Ehlanzeni in Mpumalanga (HIV prevalence = 16% in the general population), Tshwane and Ekurhuleni in Gauteng (overall HIV prevalence = 11.9% in the general population), and UMgungundlovu as well as eThekwini in KwaZulu-Natal (HIV prevalence = 17.6% and 9.2% in the general population, respectively) [32,33]. These districts were selected because the MSM programme operates within them, as they have been identified as high HIV burden areas. This selection is aligned with South African national health priorities [34]. Gauteng and KwaZulu-Natal are the most populated provinces in South Africa, with 15.9 million and 12.3 million residents, respectively, while Mpumalanga has 5.1 million residents [28,32,33,35].

### 2.4. Source of Data

The current study analysed MSM HIV programme data from 1 January 2018 to 31 December 2022. The data were collected by an NGO running the MSM programme in the five selected districts within the three provinces (Gauteng, KwaZulu-Natal, and Mpumalanga), which was funded by the Centres for Disease Control and Prevention (CDC).

#### Study Variables

Data used within this research included demographic variables such as age and district; programmatic variables such as HIV testing modality and linkage pattern; outcome variables such as ART collection as well as viral load test completion; and contextual variables such as the COVID-19 period. This study included all the above as independent variables in the multivariable models. The main outcome variable is ART re-engagement, which is defined for the current study as the process wherein individuals living with HIV return to care after being out of care for ≥60 days.

The HIV testing modality variable was included based on research showing that the type of HIV testing method can affect the support systems an individual has upon receiving their initial diagnosis. This affects how engaged the individual is with the healthcare system after receiving the initial diagnosis [21]. The linkage to care pattern was included as a proxy for initial engagement with the health system, which may affect future health-seeking behaviour and the likelihood of re-engagement following an interruption in treatment [22]. Undertaking a viral load test, regardless of the result, has been associated with increased retention in care. Viral load tests serve as an essential motivators and reinforcements when patients re-engage in care. They allow patients to see their progress towards treatment success, fast-tracking them into more convenient long-term retention programmes [36]. Hence, they were included in the current study. The variables are defined below:

- Age: The age of the participants in years at the time of data collection.
- Districts: The geographic location where the participants reside and access the programme services.
- ART status groups:
  ART-naïve: Participants who had never been on ART at the baseline of the current study.
  Continuous ART users: Participants who were already initiated on ART before the study and had been adhering to it since initiation.
  ART re-initiators: Participants who were previously initiated on ART, but had interrupted treatment for a period of ≥60 consecutive days before re-engagement.
  NB: The ART status for each participant was determined at a single point during programme enrolment. Based on the reported ART history at enrolment, participants were classified into three categories (i.e., ART-naïve, continuously on ART, and ART re-initiators). The ART status variable is a cross-sectional variable.
- HIV Testing modality: The method of HIV testing used to determine the participant’s HIV status:
  Rapid test: An HIV testing approach that uses antibodies for the detection of HIV in fluids such as blood or oral fluid, and produces results in 30 min or less.
  SNS: An HIV testing strategy that utilises network connections to refer those at high risk to HIV testing services.
  HIV index testing: An HIV testing approach wherein a newly diagnosed person living with HIV assists in identifying their undiagnosed sexual partners, biological children, and needle sharing partners, etc., to undertake voluntary HIV testing.
  HIV self-screening: A HIV testing approach wherein an individual voluntarily collects their own specimen, such as blood or oral fluid and privately conducts a rapid HIV test on themselves and interprets the results.
- Linkage pattern: Whether participants were linked for HIV only, or had other linkages (i.e., HIV and other conditions such as TB or STIs, or only the other conditions), or declined being linked altogether.
- ART collection: Whether the participants were able to collect their ART medication or not during the study period.
- Viral Load (VL) test completion: Whether the participants were able to get a viral load test or not, irrespective of the results observed.
- COVID-19 period: Whether the data were collected during the COVID-19 period (March 2020–December 2022) or prior (January 2018–February 2020).

### 2.5. Data Analysis

#### 2.5.1. Descriptive Statistics

Descriptive statistics were used to describe participant characteristics. Continuous variables were represented as mean sand standard deviations. Categorical data were represented as frequencies and percentages.

#### 2.5.2. Chi-Square Test

Chi-square tests were used to evaluate differences in categorical variables between the three ART status groups, while a one-way ANOVA was used for the continuous variable (i.e., age). The ART status was classified into three groups: ART-naive, continuous ART users, and ART re-initiators.

#### 2.5.3. Multinomial Logistic Regression

Multinomial logistic regression was used to evaluate the association between these three ART statuses (using ART-naive subjects as the reference group) as well as the association with age, district, type of HIV test used, linkage pattern to ART, viral load test completion, whether ART medication was collected, and lastly, whether data was collected during the COVID-19 period or not. Adjusted odds ratios (aOR) with 95% confidence intervals (CIs) were reported.

#### 2.5.4. Binary Logistic Regression

To evaluate the factors that influence ART re-engagement specifically, a binary logistic regression was used with participants who had previously received ART (continuing ART vs. ART re-initiation). The participants who were re-initiating their ART were coded as 1, and those continuing their ART were coded as 0. Initially, the binary logistic regression identified a quasi-complete separation in several categorical predictor variables, shown by large odds ratios. Consequently, HIV testing modalities were collapsed into rapid testing and non-rapid testing (i.e., SNS, index testing, and self-screening). Similarly, linkage patterns were collapsed into two categories based on whether a participant was linked for HIV only or other linkages/declined linkage. The Ehlanzeni district was excluded due to the absence of re-initiators in that district; findings for this district are descriptively reported in Table 1, and in the multinomial regression model (Table 2). It is acknowledged that while this approach resolved quasi-complete separation and ensured model stability, it reduces the granularity across the individual non-rapid HIV testing modalities and the specific linkage categories, limiting the precision of inference for these variables in the binary model. The COVID-19 pandemic period has been shown to disproportionately disrupt facilities’ HIV testing services compared to the community, wherein community-based HIV testing modalities exhibited higher resilience to COVID-19. The effect of COVID-19 on the relationship between HIV testing modalities (e.g., community or facility-based) and ART re-engagement was assessed by including an interaction term for both variables in the binary logistic regression model.

#### 2.5.5. Statistical Software and Significance Level

Significance was set at *p* < 0.05, and all analyses were completed using SPSS software version 31.0.1.0 (Armonk, New York, NY, USA: IBM Corp).

### 2.6. Ethical Considerations

This study was ethically approved by the University of South Africa’s College of Graduate Studies Research Ethics Review Committee (Ref no: 9698). Approval to access and analyse the routine programme data was obtained from a non-governmental organisation (Ref no: DSGC-00038).

## 3. Results

Table 1 presents the baseline characteristics of 3337 men who have sex with men stratified by ART status. The three ART groups showed comparable mean ages, i.e., 33.70, 34.35, and 33.39 years for ART-naive, continuously on ART, and ART re-initiators groups (*p* = 0.189). There was a significant difference in geographic distribution (*p* < 0.001), with 56.1% of MSM continuously on ART residing in the eThekwini district in KZN, while the ART-naive group had proportions ranging from 7.4% in Ehlanzeni (MP) to 28.9% in Ekurhuleni (GP). There were significant differences in HIV testing modalities across ART groups (*p* < 0.001). Among ART-naive MSM, HIV testing modalities included SNS (51.2%) and rapid testing (43%). Rapid testing was used among the MSM continuously on ART and ART re-initiator groups (86.9% and 71.9%, respectively). Also, ART collection rates were high across all groups (i.e., 84.3%, 91.6%, and 88.6% for ART-naive, continuously on ART and ART re-initiator groups, respectively), although the differences were statistically significant (*p* < 0.001). Viral load test completion differed significantly across groups, with completion rates of 71.4%, 58.2%, and 40.2% among the ART re-initiators, continuously on ART, and ART-naive groups, respectively. There was a significant difference in enrolment during the COVID-19 period across ART groups (*p* < 0.001), with proportions of 96.9% among ART-naive MSM, 85.9% among MSM continuously on ART, and 84.9% among the ART re-initiator group.

**Table 1:** Baseline characteristics of the men who have sex with men in the study.

Variables	ART-Naive at Baseline (n = 2640)	Continuously on ART (n = 512)	ART Re-Initiators (n = 185)	*p*-Value
Age (years) (m ± SD)	33.70 ± 7.731	34.35 ± 8.552	33.39 ± 8.093	0.189
	n(%)	n(%)	n(%)	
Districts				
City of Tshwane-GP	568 (21.5)	132 (25.8)	59 (31.9)	
Ekurhuleni-GP	762 (28.9)	52 (10.2)	35 (18.9)	<0.001
Ehlanzeni-MP	196 (7.4)	4 (0.8)	0 (0)	
EThekwini-KZN	654 (24.8)	287 (56.1)	44 (23.8)	
UMgungudlovu-KZN	460 (17.4)	37 (7.2)	47 (25.4)	
HIV Testing modality				
Rapid test	1136 (43.0)	445 (86.9)	133 (71.9)	
HIV self-screening	75 (2.8)	2 (0.4)	2 (1.1)	<0.001
SNS	1351 (51.2)	64 (12.5)	49 (26.5)	
Index testing	78 (3.0)	1 (0.2)	1 (0.5)	
Linkage pattern				
Linked for HIV only	2605 (98.7)	501 (97.9)	183 (98.9)	
Declined linkage	11 (0.4)	0 (0)	0 (0)	0.022
Linked to HIV and TB	16 (0.6)	11 (2.1)	1 (0.5)	
Other (linked for STI or TB only)	8 (0.3)	0 (0)	1 (0.5)	
ART collection				
No	415 (15.7)	43 (8.4)	21 (11.4)	<0.001
Yes	2225 (84.3)	164 (91.6)	164 (88.6)	
Viral load test completion				
No	1577 (59.8)	214 (41.8)	53 (28.6)	<0.001
Yes	1061 (40.2)	298 (58.2)	132 (71.4)	
COVID-19 period				
No	83 (3.1)	72 (14.1)	28 (15.1)	<0.001
Yes	2557 (96.9)	440 (85.9)	157 (84.9)	

n: sample size; SD: Standard Deviation; m = mean; ART: Antiretroviral therapy; TB: Tuberculosis; STI: Sexually Transmitted Infections; GP: Gauteng Province; MP: Mpumalanga Province; KZN: KwaZulu-Natal Province; COVID-19: Coronavirus disease 2019; SNS: Social Network Strategy.

Table 2 presents the multivariable logistic regression showing the factors associated with ART status (i.e., the ART-naïve and ART re-initiator groups, with those continuously on ART as the reference category). There was a significant association between MSM who were ART-naive at baseline and HIV testing modality (aOR = 2.831, 95% CI: 2.458–3.261, *p* < 0.001). The odds of being ART-naive substantially increased with being enrolled during the COVID-19 period (aOR = 3.583, 95% CI: 2.483–5.170, *p* < 0.001). On the other hand, ART collection (aOR = 0.413, 95% CI: 0.290–0.588, *p* < 0.001), district (aOR = 0.834, 95% CI: 0.776–0.897, *p* < 0.001), and linkage pattern (aOR = 0.693, 95% CI: 0.481–0.997, *p* = 0.048) were significantly associated with a lower likelihood of being ART-naive. There was no significant association observed with age and viral load test completion among ART-naïve MSM (aOR = 0.993, 95% CI: 0.980–1.005, *p* = 0.237). Additionally, there was a significant association between being re-initiated on ART and HIV testing modality (aOR = 1.845, 95% CI: 1.485–2.293, *p* < 0.001), although the effect was smaller compared to that of the ART-naive group (aOR = 2.831, 95% CI: 2.458–3.261, *p* < 0.001). There was a significant positive association observed between viral load test completion and ART re-initiation (aOR = 2.238, 95% CI: 1.529–3.275, *p* < 0.001). There was no association found between ART re-initiation and the COVID-19 period (aOR = 1.037, 95% CI: 0.615–1.751, *p* = 0.890), linkage pattern (aOR = 0.802, 95% CI: 0.416–1.546, *p* = 0.510), district (aOR = 0.915, 95% CI = 0.811–1.033, *p* = 0.153), or ART collection (aOR = 0.702, 95% CI = 0.398–1.239, *p* = 0.223).

**Table 2:** Multinomial logistic regression of factors associated with antiretroviral therapy status (ART-naive, continuous ART use, ART re-initiation) among men who have sex with men.

ART Status		Df	aOR	95% CI		
				Lower Bound	Upper Bound	*p*-Value
ART-naive at baseline	Age (years)	1	0.993	0.980	1.005	0.237
	District	1	0.834	0.776	0.897	<0.001
	HIV testing modality	1	2.831	2.458	3.261	<0.001
	Linkage pattern	1	0.693	0.481	0.997	0.048
	ART collection	1	0.413	0.290	0.588	<0.001
	Viral load test completion	1	0.841	0.681	1.039	0.109
	COVID-19 period	1	3.583	2.483	5.170	<0.001
ART re-initiators	Age (years)	1	0.987	0.966	1.008	0.210
	District	1	0.915	0.811	1.033	0.153
	HIV testing modality	1	1.845	1.485	2.293	<0.001
	Linkage pattern	1	0.802	0.416	1.546	0.510
	ART collection	1	0.702	0.398	1.239	0.223
	Viral load test completion	1	2.238	1.529	3.275	<0.001
	COVID-19 period	1	1.037	0.615	1.751	0.890

Ref: Continuously on ART; ART: Antiretroviral therapy; Coronavirus disease 2019; CI: Confidence Interval; aOR: Adjusted Odds Ratios; df: Degrees of Freedom.

Table 3 below presents the binary logistic regression showcasing the factors associated with re-engagement among MSM who were previously initiated on ART. District was the only statistically significant predictor of re-engagement among MSM in the current study, wherein the eThekwini district had lower odds of re-engagement (aOR = 0.248, 95% CI: 0.144–0.428, *p* < 0.001). This represented a 75% reduction in the likelihood of re-engagement when compared to the City of Tshwane as the reference group. No significant associations were observed with any of the other predictors, including age (*p* = 0.562), HIV testing modality (*p* = 0.127), ART collection (*p* = 0.322), linkage pattern (*p* = 0.996), the COVID-19 period (*p* = 0.404), viral load test completion (*p* = 0.096), and the interaction effect of the COVID-19 period with HIV testing modality (*p* = 0.619).

## 4. Discussion

This study aimed to identify factors associated with ART re-engagement among MSM in selected districts of South African provinces. District was identified as the only significant (*p* < 0.001) predictor of re-engagement, wherein MSM from the eThekwini district had significantly lower odds of re-engaging with care. This corroborates previous studies reporting that geographic factors predominantly predicted re-engagement with care more than individual demographic characteristics [14,18,31].

Although HIV testing modality was significantly associated with being re-initiated on ART in the multinomial regression of the current study, it was not found to be a predictor of re-engagement in the binary regression among previously initiated MSM. This is inconsistent with findings by Sebati and Brown [30], who demonstrated a significant association from a binary regression model between HIV testing modality and ART initiation among MSM from the same programme and within the same South African districts as the current study. This may be due to the differences in the definitions of the outcomes (ART initiation vs. ART re-initiation) and analytic approaches. Nonetheless, ART-naïve MSM were mostly found to be tested through the SNS, a community- and peer-led testing approach wherein trained peer recruiters from existing networks refer individuals at high risk of HIV for testing and care outside of formal health facility settings. However, both MSM who were continuously on ART and those who re-initiated ART were mostly tested using rapid testing, which is a health-facility-based HIV testing approach. Seemingly, rapid testing captures MSM who were already engaging in care through formal health facilities, while SNS reaches MSM in their communities and social spaces. The ART-naive MSM are likely to be the most disconnected from formal health facilities, making community- and peer-led testing approaches more effective at reaching this group, particularly at first diagnosis/engagement. This demonstrates the importance of HIV testing modality to an individual’s HIV care continuum trajectory from diagnosis to engagement, and from re-engagement to care [37]. This is particularly relevant given that ART and viral suppression rates for MSM continue to lag behind UNAIDS 95-95-95 goals, with approximately 67–78% of MSM living with HIV in eastern, southern, central, and western Africa being on ART, and 69% virally suppressed as of 2020 [38]. This underscores the need for combination interventions designed specifically for MSM to address geographical, social, structural, and behavioural vulnerabilities [38].

The lack of a significant association between HIV testing modality and ART re-engagement in the binary logistic regression model suggests that an individual’s initial mode of identification and HIV testing becomes a less relevant predictor of re-engagement when prior ART experience has been established [39]. Rather, district emerged as a significant predictor of re-engagement among ART-experienced MSM in the current study, suggesting programme-level and geographic factors at the point of return to care are more proximal determinants than HIV testing modality. The collapsing of non-rapid testing modalities into one category in the binary model limits the ability to draw conclusions about the differences between HIV self-screening, SNS and index testing concerning re-engagement with care. The relationship between specific HIV testing modalities and ART re-engagement, therefore, presents an important area for future investigations [40].

The MSM who re-initiated ART in the current study had the highest rates of viral load test completion compared to ART-naive individuals and those who were continuously on ART. This may indicate that these individuals maintained their motivation to seek out healthcare when they returned, and suggests that those who returned to care are clinically motivated and proactive in monitoring their treatment outcomes. A study by Kitenge et al. [29] observed an increase in the number of ART-experienced individuals presenting to a rural area of KwaZulu-Natal with advanced disease. They noted that the health-seeking behaviour of these men is often driven by their current state of health, rather than by regular procedures for monitoring health. Likewise, a relatively high proportion of ART re-initiators have completed viral load testing within the current study, which could suggest that the reason for re-engaging with the health system is clinical need rather than routine use of the healthcare system [41].

This study did not find any association or interaction between ART re-initiation/re-engagement and the COVID-19 period. This may point to the resilience of the MSM programme’s ability to continue providing services throughout the COVID-19 pandemic. Possibly, the pandemic phase may have contributed to the strengthening of structural re-engagement opportunities, including expanded options for differentiated service delivery models [42]. A scoping review of the literature conducted by Magura et al. [43] noted that COVID-19 impacted adherence to ART because of increased economic and social vulnerabilities and fragmented service delivery. They reported that local community adaptive strategies (e.g., using peer-to-peer distribution networks and the increased use of telemedicine) helped mitigate some of these impacts [43].

District was the only significant predictor of ART re-engagement in the binary logistic regression model of the current study, wherein the eThekwini district in KwaZulu-Natal had lower odds of re-engaging with care compared to the City of Tshwane in Gauteng (aOR = 0.248, 95% CI: 0.144–0.428, *p* < 0.001). Descriptively, eThekwini had a notably large proportion of MSM who were continuously on ART (56.1%), yet the lowest odds of re-engagement among those who had previously experienced ART interruptions. This points to meaningful variations between districts, which were not explained by any other programme-level factors included in the study. Two studies conducted by Hannaford et al. [44] and Modipane et al. [45] have highlighted distance-related barriers, such as the difficulty in accessing ART while travelling for work, as well as financial constraints associated with travelling to locations far from home. Bono et al. [46] support the notion that geography is an important, yet often unrecognised, factor affecting the continuity of HIV care. Variations between the eThekwini and Tshwane metropolitan regions may reflect differences in both the density and quality of services available to MSM, as well as the social networks and community support structures that exist within each city, which may facilitate MSM re-engaging with HIV care. While not directly included in the current analysis, these factors present an important area for future qualitative research.

District, as included in the current analysis, may serve as a macro-level representation of geographic, programmatic, and service delivery variations between metropolitan areas. However, as a single measure, it cannot provide insight into each factor independently. The lack of individual-level variables, i.e., stigma experience, mental health, socioeconomic status, and healthcare-provider attitudes, in these routine programme data creates an omitted variable bias whereby there may be unmeasured socio-structural characteristics that differ systematically by district and partly contribute to the observed geographic variations in re-engagement.

## 5. Strengths and Limitations

This study has several strengths. First, it uses a large, multi-district, routine programme dataset that covers five districts in three provinces, providing the opportunity to conduct a geographically diverse analysis of ART re-engagement for MSM. This is a population that is very difficult to study due to high levels of stigma, criminalisation, and under-representation in routine health information systems. Second, the multi-year programme data allowed for the classification of MSM into different ART status categories, thus allowing for a comparative analysis of the predictors associated with re-engagement. Third, the application of multinomial and binary logistic regression models provides the capability to rigorously adjust for potential confounding variables, with analytical decisions transparently reported. Fourthly, this study adds to the limited body of literature on ART re-engagement for MSM in South Africa, which represents a key and often underserved population in the HIV response [14,15].

Similarly, this study has some limitations. This study analysed routine programme data; hence has the inherent limitations of using secondary data, including potential data quality challenges and missing data and the absence of some key variables is acknowledged. Individual-level factors that may contribute to re-engagement outcomes, i.e., stigma experiences, mental health status, substance use, socioeconomic status and healthcare provider attitudes, etc., were not captured in the routine data and hence could not be included in the current analysis. The ART status for each participant was determined at one point during programme enrolment; hence individual-level changes in ART status were not tracked longitudinally, preventing causal inference. Findings from this study may have limited generalisability to MSM in non-structured, NGO-led HIV programmes. As such, the possibility of selection bias cannot be excluded from the study since only programme-enrolled MSM were included. From an analytical standpoint, quasi-complete separation in the binary logistic regression necessitated the collapsing of HIV testing modality into binary categories (rapid vs. non-rapid HIV testing). Similarly, the linkage pattern was collapsed to a binary variable (i.e., linked to HIV only vs. other linkage). While this approach is statistically acceptable and ensures statistical stability, it obscures heterogeneity across the individual non-rapid HIV testing modalities (i.e., HIV self-screening, SNS, and index testing), which may have a differential association with re-engagement with care that the current analysis could not explore. In the same way, the exclusion of the Ehlanzeni district from the binary logistic regression analysis due to a lack of ART re-initiators limited district-level inference in this model. Ehlanzeni district findings are therefore descriptively retained in Table 1 and the multinomial regression model (Table 2). Future studies with larger samples of ART re-initiators across all districts and HIV testing modalities would allow for a more granular analysis of these specific associations.

## 6. Conclusions

This study identified the district as the only statistically significant predictor of re-engagement to care among MSM who re-initiated ART, with MSM from the eThekwini district having lower odds of re-engagement to care compared to those in the City of Tshwane. In the multinomial logistic regression model, HIV testing modality was significantly associated with ART status, with SNS mainly identifying ART-naive MSM. However, it was not significantly associated with re-engagement in the binary regression model. The results suggest that geographic and programme-level factors at the time a person is returning to care as being more proximal determinants of an individual’s re-engagement to care than the mode of HIV test they initially accessed. The district variable likely serves as a high-level proxy for the combination of programme reach, service density and unmeasured socio-structural factors that vary across metropolitan areas. Future studies should focus on the qualitative assessment of programme-specific and contextual factors that are driving the district variability of re-engagement, and must also include individual-level variables not captured in routine data. A longitudinal analysis of ART status transitions captured over time is also necessary to provide greater strength of evidence for causal inference.

## Figures and Tables

**Table 3:** Binary logistic regression showing the factors associated with re-engagement among previously initiated MSM.

	Df	aOR	95% CI		*p*-Value
			Lower	Upper	
Age (years)	1	0.994	0.972	1.016	0.562
Districts	3	-	-	-	<0.001
City of Tshwane, GP (ref)	-	-	-	-	-
Ekurhuleni-GP	1	1.103	0.617	1.973	0.741
Ethekwini-KZN	1	0.248	0.144	0.428	<0.001
UMgungudlovu-KZN	1	1.776	0.972	3.247	0.062
HIV testing modality	-	-	-	-	0.127
Ref- Rapid HIV test (ref)	-	-	-	-	-
Non-rapid HIV test	1	7.299	0.567	94.037	0.127
Linkage pattern	-	-	-	-	0.996
Linked for HIV only (ref)	-	-	-	-	-
Declined/other linkage	1	1.004	0.196	5.150	0.996
ART collection	-	-	-	-	0.322
No (ref)	-	-	-	-	-
Yes	1	0.740	0.408	1.343	0.322
Viral load test completion	-	-	-	-	0.096
No (ref)	-	-	-	-	-
Yes	1	1.441	0.937	2.218	0.096
COVID-19 period		-	-	-	0.404
No (ref)	-	-	-	-	-
Yes	1	1.864	0.431	8.051	0.404
Interaction effect	-	-	-	-	-
COVID-19 period X HIV testing modality	1	0.722	0.200	2.607	0.619

CI: Confidence Interval; aOR: Adjusted Odds Ratios; df: Degrees of Freedom ART: Antiretroviral therapy; GP: Gauteng Province; KZN: KwaZulu-Natal Province; COVID-19: Coronavirus disease 2019; Ehlanzeni district excluded due to 0 re-initiators; HIV testing modalities and linkage were re-grouped into binary variables, i.e., rapid HIV testing vs. non-rapid HIV testing, and linked to HIV only vs. declined/other linkage.

## Data Availability

Data are available upon request from the corresponding author.

## Abbreviations

The following abbreviations are used in this manuscript:

HIV	Human Immunodeficiency Virus
ART	Antiretroviral therapy
MSM	Men who have Sex with Men
SNS	Social Network Strategy
NGO	Non-governmental Organisation
PLHIV	People living with HIV
CDC	Centres for Disease Control and Prevention
